# The Protective Effects of Insulin and Natural Honey against Hippocampal Cell Death in Streptozotocin-Induced Diabetic Rats

**DOI:** 10.1155/2014/491571

**Published:** 2014-03-13

**Authors:** Iraj Jafari Anarkooli, Hossein Barzegar Ganji, Maryam Pourheidar

**Affiliations:** ^1^Department of Anatomy, Faculty of Medicine, Zanjan University of Medical Sciences, Zanjan 4513956111, Iran; ^2^Department of Histology, Faculty of Medicine, Urmia University of Medical Sciences, Urmia, Iran

## Abstract

We investigated the effects of insulin and honey as antioxidants to prevent the hippocampal cell death in streptozotocin-induced diabetic rats. We selected sixty Wister rats (5 groups of 12 animals each), including the control group (C), and four diabetic groups (control (D) and 3 groups treated with insulin (I), honey (H), and insulin plus honey (I + H)). Diabetes was induced by streptozotocin injection (IP, 60 mg/kg). Six weeks after the induction of diabetes, the group I received insulin (3-4 U/kg/day, SC), group H received honey (5 mg/kg/day, IP), and group I + H received a combination of the above at the same dose. Groups C and D received normal saline. Two weeks after treatment, rats were sacrificed and the hippocampus was extracted. Neuronal cell death in the hippocampal region was examined using trypan blue assay, “H & E” staining, and TUNEL assay. Cell viability assessment showed significantly lower number of living cells in group D than in group C. Besides, the mean number of living cells was significantly higher in group I, H, and I + H compared to group D. Therefore, it can be concluded that the treatment of the diabetic rats with insulin, honey, and a combination of insulin and honey can prevent neuronal cell death in different hippocampal areas of the studied samples.

## 1. Introduction

Diabetes is one of the most common metabolic disorders in humans. Long-term diabetes is a high risk factor for developing microvascular complications such as retinopathy, nephropathy, and neuropathy [1, 2]. Numerous studies pertaining to diabetes-induced neural dysfunction have been reported. These studies have mainly explored the functions of the peripheral nervous system. However, evidence from recent research has indicated that the central nervous system may also be involved in diabetes [1, 3–5]. Hippocampus forms a part of the limbic system that is associated with learning and memory formation in animals as well as in humans [6]. The hippocampus contains high density of insulin receptors and insulin-sensitive transporters [7]. Insulin receptors in the brain are also associated with cognitive functions and play a crucial role in neurodegenerative diseases such as Alzheimer and Parkinson [8, 9]. Considering the significance of hippocampus in neurodegenerative diseases and its susceptibility to metabolic disorders, the hippocampus can be regarded as one of the most sensitive regions of the central nervous system to be associated with diabetes [10].

In diabetic encephalopathy, which is characterized by cognitive and memory impairment, changes in the hippocampus have been observed, but the mechanism that triggers these changes is unknown [2]. Findings from recent research suggest that dendritic atrophy, decrease in the number of glucocorticoid receptors, change in expression of insulin growth factor, and induction of apoptosis may be responsible for these changes [1, 11]. Apoptosis is a form of cell death that is determined by the fragmentation of DNA in the cell nucleus by using the TUNEL assay, which is undoubtedly one of the techniques used for detection of cell death induced by apoptosis [12–14]. Furthermore, studies have reported electrophysiological and neuroanatomical changes in Streptozotocin (STZ-) induced diabetic rats. Similarly, other studies have indicated the simplification of dendritic tree as well as the neuronal death in the hippocampus of STZ-induced diabetic rats [15].

Another study has shown an increase in oxidative stress particularly at CA3 and dentate gyrus regions of the hippocampus in STZ-induced diabetic rats [16]. The increase in blood sugar level due to diabetes followed by subsequent increase in oxidative stress leads to the formation and accumulation of excess free radicals and reactive oxygen species (ROS). The free radicals and ROS are responsible for neuronal cell death [17].

Studies have shown that treatment with antioxidants reduces some of the complications arising from diabetes [18]. In addition, researchers have revealed that honey can be used as an antioxidant to protect various organs including the brain and the heart from oxidative damages [19–21]. Although, exactly how honey exhibits its protective effect is not known, recent research findings have demonstrated that honey plays a role in reducing cell death resulting from oxidative stress, as well as reduction of apoptosis in rats' testicular germ cells [22–24]. However, not much information is available about the effect of controlling blood sugar levels and the use of honey as an antioxidant in preventing cell death under diabetic condition. To the best of our knowledge, the present study is the first to evaluate the effects of insulin, honey, and the combination of the two in preventing hippocampal cell death in STZ-induced diabetic rats.

## 2. Methodology

The experimental study was conducted on 60 male Wistar rats (8–10 weeks old) and weighing approximately 200–250 g. Animals were purchased from Razi Institute in Tehran, and were transferred to Zanjan University of Medical Sciences. During the study period, the animals had free access to food and water and the animal house was maintained on an inverted 12 h light-dark cycle at the University of Medical Sciences. All procedures were in accordance with the Guide for the Care and Use of Laboratory Animals of Zanjan University of Medical Sciences, Iran. Before the beginning of the actual experiment, the weight of each animal was recorded and blood was drawn from the caudal vein for measuring blood glucose level by using a glucometer (Accu-chek, Germany). The rats were then allowed to fast for 24 h. After 24 h of fasting, the animals were anesthetized by applying chloroform and STZ (Sigma, USA) was injected intraperitoneally (IP) at 60 mg/kg for the induction of diabetes [25]. The injection solution was prepared by dissolving STZ in physiological saline. The control group was selected to receive normal saline in order to induce stress as a result of injection. The STZ injection day was assigned as day zero and three days (72 h) later, the blood glucose level of the diabetic rats were recorded. Rats with blood glucose level above 250 mg/dL were considered diabetic [26] and rats with blood glucose level below 250 mg/dL were excluded from this study. At the end of the sixth week, STZ-induced diabetic rats were randomly divided into the following four groups, (each group consisted of 12 rats).

1. The diabetic group that received no treatment (D).
2. The diabetic group treated with insulin (I); rats belonging to this group received subcutaneous injections of protamine insulin (NPH) (Exir Boroojerd Pharmacy, Iran). The dose administered was between 3 to 4 units per day.
3. The diabetic group treated with natural honey (H) (Natural Food Industries of Keshtzar-e Sabz Khansar, Iran); rats in this group received intraperitoneally (IP) daily doses of natural honey (5 mg per kg of body weight) diluted in distilled water [27, 28].
4. The diabetic group treated simultaneously with insulin and natural honey (I + H); the daily doses of insulin and natural honey were the same as the doses mentioned in “2” and “3,” respectively.

Apart from these four groups, another control group (C) was formed with rats who had not received STZ. Groups C and D received equal amounts of normal saline. The treatment regimen was continued for 2 weeks, and at the end of the treatment period (end of eighth week counting from day zero), the blood sugar levels and the body weights of rats from each group were recorded. After completion of the final measurements, rats were anesthetized with the application of chloroform and their brains were surgically removed by opening the skull along the midline. The right and the left hippocampus inside the brain were isolated by pulling off the meninges carefully with the help of a forcep. The right hippocampus was used in the histopathological studies and the TUNEL assay, and the left hippocampus was frozen and stored in liquid nitrogen for the purpose of cell counting.

### 2.1. Preparation of Honey for Injection

To prepare the honey for injection, 20 g of pure natural honey was added into 20 mL of distilled water, and a homogenous solution was obtained by continuous mixing and shaking with the help of a machine. The concentration of honey in the final solution was 1 g/mL and this solution was stored at room temperature. The weight of each rat was recorded prior to injection, and the final concentration of the injection solution was adjusted according to the body weight of each rat (5 g per kg of rat body weight). The injection was given intraperitoneally to each rat in the desired group.

### 2.2. Assessment of Cell Viability Using Trypan Blue Staining

The cell sediment was broken with the DMEM/F_12_ solution and the initial volume was determined. On the other hand, 0.4 g of trypan blue was dissolved in 100 mL of normal saline, and the undissolved particles were filtered using a 0.22 *μ*m filter. A total of 100 mL of cell suspension and 100 mL of trypan blue solution were mixed for cell counting. The mixture solution was layered on the two edges of the counting slide covered with a coverslip and the liquid was drawn into the void by the mechanism of capillary action. To get an accurate count, no pressure was applied to discharge the fluid by Pasteur pipette. The two halves of the slide were filled with cell fluid and the cells were counted separately by two different people. The cells that took the blue stain were considered as dead cells and the colourless cells were considered as viable cells. The cell counting was done using an Olympus BX51 light microscope (Japan) equipped with a camera.

### 2.3. Histopathological Study

The hippocampus samples obtained from rats were immersed in 10% formalin from 48 to 72 h to allow fixation. For tissue processing after fixation, slow step-wise dehydration was carried out by adding a series of alcohol solutions of increasing concentration, and each time prior to the addition of new solution, the samples were washed with xylene. After dehydration, the hippocampus samples were embedded in melt paraffin. Sectioning was performed by using the rotary microtome from which slices of thickness 5 *μ*m were obtained. The slices were mounted on a microscopic slide and any paraffin adhering to the mounted sections was dissolved by passing Clearene. Hematoxylin and eosin stains (abbreviated as “H & E”) were used for staining the sections. The slides were observed under a light microscope (Olympus BX51), and clear images of the slides were captured by the camera fitted with the microscope. A total of 5 photomicrographs corresponding to 400x magnification were prepared from each sample, out of which 4 photomicrographs were chosen randomly for counting cells at the hippocampal CA3 region by Digital Imaging Solution Cell Software.

### 2.4. Quantification of Apoptosis Using TUNEL Assay

The TUNEL assay was first reported by Gavrieli et al., in 1992 [29], and since then, it has been widely used for detecting apoptotic cells in situ. The assay relies on the ability of the enzyme terminal deoxynucleotidyl transferase (TdT) to catalyze the addition of dUTP to the 3′-OH end of the fragmented DNA. The enzyme would also add labeled nucleotides such as DIG-dUTP, biotin-dUTP, and fluorescein-dUTP to the 3′ terminus of a DNA molecule. If labelled Avidin with peroxidase attached to biotin is used, the labeled cells can be identified under light microscopy. After sectioning of the rat hippocampus, coronal slices of thickness 5 micron were transferred at a distance of 2.5 to 3 mm posterior to Bregma on silanated slides. For identifying the apoptotic cells, the TUNEL staining was done according to the manufacturer's instruction provided with the TUNEL kit (Roche, Germany), with some modifications [30]. Briefly, the tissue sections were dewaxed and rehydrated by heating at 60°C, followed by washing with xylene and rehydration with ethanol and double-distilled water. Then, the sections were incubated for 30 min at 37°C in proteinase K working solution (20 *μ*g/mL in 10 mmol/L Tris-Cl, pH 7.6). The sections were washed with PBS (twice for 5 min each) and incubated with the TUNEL reaction mixture for 1 h at 37°C inside a humidified chamber. After converter peroxidase was added, the slices were incubated for 30 min at 37°C in the humidified chamber. Then, 3,3-diaminobenzidine (DAB) substrate was added for the observation of nuclei with DNA nick-end labeling (in the dark for 10 min). The sections were counter-stained with hematoxylin for 2 min. 5 photomicrographs corresponding to 400x magnification were prepared from each sample, out of which 4 photomicrographs were chosen randomly. The number of TUNEL-positive cells in the hippocampal region was counted by double-blinded observation, analyzed by Digital Imaging Solution Cell Software, and pictured by light microscope (Olympus BX51) equipped with a camera.

### 2.5. Statistical Analyses

All data were expressed as Mean ± SD. The software SPSS (version 16) was used for data analysis. The data was analyzed by performing (ANOVA) and (post hoc) tests and *P* ≤ 0.05 was considered as statistically significant.

## 3. Results

### 3.1. Blood Glucose and Body Weight Changes

The blood sugar measurements in diabetic rats after a 2-week treatment period showed significant variations among different groups (*P* < 0.01). The blood glucose levels in diabetic rats treated with honey H were reduced, but the difference between the reduced value and the blood sugar level of the untreated diabetic group D was not significant (*P* = 0.4). The blood sugar levels in groups D and H were significantly higher (*P* < 0.01) than those in other groups. No significant differences in blood glucose levels were observed among insulin treated group I, the group treated with insulin and honey I + H, and the nondiabetic control group C (Table 1). At the beginning of the study, all five groups had almost identical weights, but after 6 weeks, the weights of the diabetic rats were decreased considerably compared to the control group C (*P* < 0.01). In addition, at the end of the treatment period, the weights of the diabetic groups I and I + H were significantly increased (*P* < 0.01) compared with group D. There were no significant differences in weights between groups H and D and between groups I and I + H (*P* = 0.8 and *P* = 0.2, resp.) (see Table 1).

### 3.2. The Results of Cell Count and Viability Assay

The cell membrane of a dead cell is permeable to trypan blue, whereas the membrane of a live cell is impermeable and does not take the blue stain of trypan blue. Therefore, trypan blue was used to selectively color dead cells blue. Under the microscope, the colorless live cells looked small, round, and refractile while the nonliving cells appeared swollen, big, and dark blue. Cell counts in the left hippocampus region showed that the average population density of live cells was significantly higher in diabetes treated groups I, H, and I + H than that of the diabetic group D, which received no treatment (*P* < 0.001). However, the average survival rate of live cells in the control group C was comparable to that of the treated groups I, H, and I + H as shown in (Table 2).

### 3.3. Histopathological Assessment

**Figure 1:**
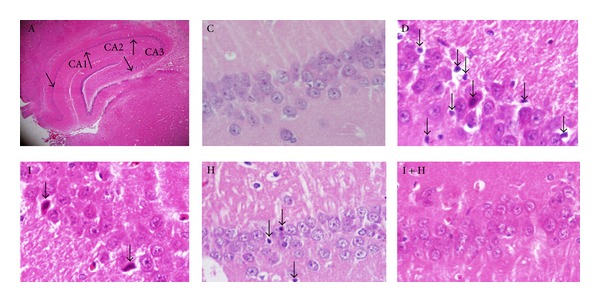
The effects of insulin and honey on neural cell death of hippocampus. Neuronal cell death was evaluated via hematoxylin-eosin staining at 400x magnification (arrows show the dead neuronal cells). A: different parts of hippocampus; C: the control group; D: untreated diabetic group; I: diabetic rats treated with insulin; H: diabetic rats treated with honey; I + H: diabetic rats treated with insulin and honey simultaneously.

**Figure 2:**
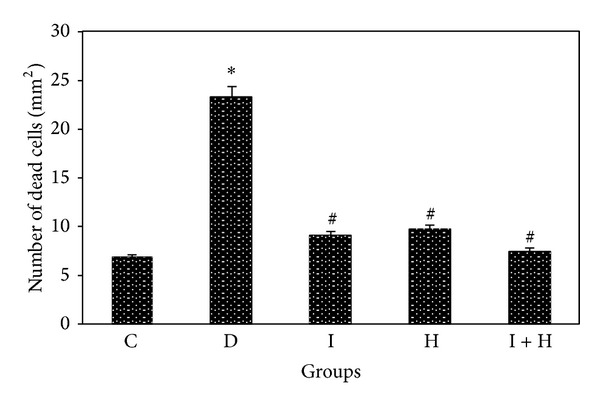
The effects of insulin and honey on neuronal cell death in the hippocampus of rats in each group (12 rats in each group). Bar graph indicates the mean ± SD *P < 0.0001 versus control group (C); ^#^ *P* < 0.001 versus diabetic group (D).

The hematoxylin-eosin staining technique was used to stain the nucleus and cytoplasm of cells. The nucleus and cytoplasm appeared blue and pink, respectively, under “H & E” staining, as shown in (Figure 1). Apoptotic cells appeared smaller than normal cells and were characterized by eosinophilic cytoplasm and pycnotic nucleus. These cells were ingested by neighbouring cells. Therefore, they could be seen in cells adjacent to the vacuoles that were formed to phagocyte the apoptotic cells. Dead or nonviable cells have been indicated with arrows in the hippocampus of untreated diabetic rats D, diabetic rats treated with insulin I, honey H, and a combination of insulin and honey simultaneously I + H. As depicted in (Figure 2), the number of dead cells in the hippocampus of diabetic rats without treatment D was significantly higher than the control group C (*P* < 0.0001). The results also showed that the number of dead cells was significantly lower in the treatment groups I, H, I + H than the untreated group D (*P* < 0.001) (Figure 2).

### 3.4. The Results of the TUNEL Assay

**Figure 3:**
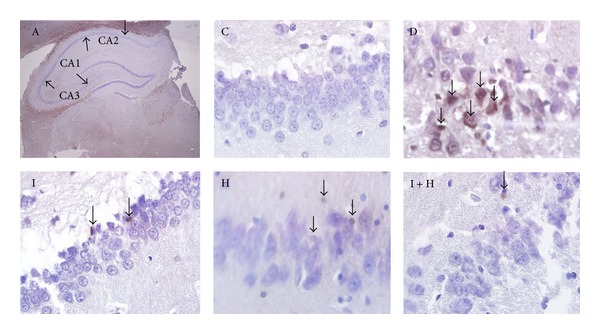
Effects of insulin and honey on apoptosis of neuronal cells in the hippocampus. Apoptosis was evaluated via TUNEL assay with the use of in situ Cell Death Detection Kit at 400x magnification (arrows show the apoptotic cells). A: different parts of hippocampus; C: the control group; D: untreated diabetic group; I: diabetic rats treated with insulin; H: diabetic group treated with honey; I + H: diabetic rats treated with insulin and honey simultaneously.

**Figure 4:**
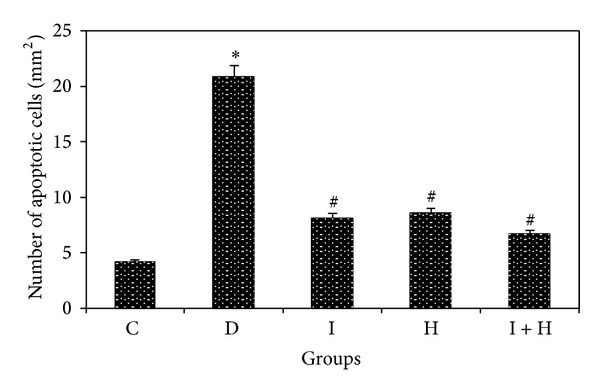
Effects of insulin and honey on apoptosis of neuronal cells in the hippocampus of rats in each group (12 rats in each group). Bar graph indicates the mean ± SD. ^#^ *P* < 0.0001; *P ≤ 0.01 compared to the control group C; ^#^ *P* < 0.001 compared to the diabetic group D.

In the TUNEL assay, the apoptotic cells in the hippocampus were distinctly identified as brown stains in the midst of blue background corresponding to the blue stains of nonapoptotic cells (Figure 3). The bar graphs in (Figure 4) revealed that apoptosis had occurred to a significant extent in the hippocampus (CA3) of untreated diabetic rats D in comparison to the control group C (*P* < 0.0001). In contrast, the diabetic rats in groups I, H, and I + H who underwent treatment for 2 weeks with insulin, honey, and a combination of insulin and honey showed significantly fewer numbers of apoptotic cells (*P* < 0.001). Notably, the differences in the number of apoptotic cells among the control group C and the treatment groups I, H, and I + H were not statistically significant.

## 4. Discussion

The results from this study have shown that hyperglycemia induced by injecting STZ in rats leads to cell death of neuronal cells in the hippocampus. The control of blood glucose levels in the diabetic rats, who underwent treatment with either insulin or honey or combination of honey and insulin could effectively prevent the progression of neuronal damage in the hippocampus as evident from the experimental fact that the number of apoptotic cells in rats undergoing treatment for diabetes was significantly lower than that of the untreated diabetic group. The detrimental effect of diabetes on the neuronal cells of hippocampus was assessed by determining the number of cells undergoing apoptosis, which was identified from the TUNEL assay. In a similar study conducted by Li et al., the hippocampus of diabetes-prone transgenic BB/W rats was examined by using the TUNEL method. The results from this study showed that after eight months of diabetes, apoptosis was enhanced in the transgenic rats, but not in the control rats [31]. Other researchers have detected, with the help of electron microscopy, the neuronal cell death in the hippocampus of STZ-induced diabetic rats after 21 days following STZ injection [14]. Zhou et al. reported neuronal cell death particularly at the CA3 and dentate gyrus regions of the hippocampus in STZ-induced diabetic rats. This neuronal cell death was observed under an electron microscope 4 weeks after the initiation of diabetes [32]. In addition, Jafari Anarkooli et al., studied the role of proapoptotic (Bax) and antiapoptotic (Bcl-2 and Bcl-X_L_) genes by using RT-PCR and Western Blotting techniques, and monitored the activity of caspase-3 through DNA-Laddering technique. All these studies support the fact that the diabetes induced by STZ is capable of inducing apoptosis in the hippocampus of rats [33]. Therefore, findings from this study have been consistent with findings from reported research studies. The mechanism that resulted in the death of neurons from hyperglycemia has not yet been established. However it is likely that ROS is responsible for cell death. As a result, it can be hypothesized that hyperglycemia stimulates an increase in the production of ROS by a mechanism that induces the activation of MAP kinase signaling pathways and the activated kinase proteins may play a major role in apoptosis [17, 34].

The study results showed that glycemic control by insulin in insulin-treated diabetic groups inhibited apoptosis in the hippocampus of STZ-induced diabetic rats. A similar study performed by Srinivasan et al. demonstrated that the number of apoptotic cells in the Dorsal root ganglion of STZ-induced diabetic rats was significantly reduced in response to treatment with insulin for 2 weeks, whereas the diabetic rats who did not receive any treatment showed no such reduction [34]. In another study by Li et al., it was found that C-peptide, a derivative of insulin, had similar effects on the hippocampus of BB/W transgenic rats [31, 35]. Furthermore, in another study, apoptosis of cortical neurons in STZ-induced diabetic rats was controlled with insulin prior to ischemia in the middle cerebral artery [36]. Jafari Anarkooli et al., also showed that insulin inhibited apoptosis in the hippocampus of STZ-induced diabetic rats by decreasing the expression of Bax proapoptotic gene and increasing the expressions of Bcl-2 and Bcl-X_L_ antiapoptotic genes, as well as by decreasing caspase-3 activity [37]. Also, consistent with this study, previous studies showed that insulin prevented apoptosis of oligodendrocytes [38]. In addition, the antiapoptotic role of insulin in nonneural structures was reported; insulin reduced apoptosis in renal tubular cells of STZ-induced diabetic rats [39]. In another study, insulin prevented apoptosis in epithelial and cancer cells [40, 41]. The results from all these studies demonstrated the protective role of insulin in preventing apoptosis of neuronal as well as other cell types. The inhibition of programmed cell death can be influenced by different factors, such as neurotrophins, synthesis of growth factors, regulation in the expressions of apoptotic modulatory proteins, and stabilization of blood sugar by the use of insulin, which as a neurotrophic factor can protect neurons against cell damage [42]. Findings from previous research have also indicated that insulin, proinsulin, C-peptide, and insulin-like factors have antiapoptotic effects [43, 44]; hence, insulin plays a dual role of stabilizing blood sugar level as well as inhibiting apoptosis [45]. Insulin exerts its antiapoptotic effect through complex mechanisms involving multiple signaling pathways resulting in increased expressions of antiapoptotic genes, as well as by protecting the mitochondrial membrane integrity against free radicals [46]. This study shows that the use of honey as an antioxidant can effectively inhibit apoptosis or neuronal cell death in the hippocampus of STZ-induced diabetic rats. The study findings are consistent with findings from previous research report in which it has been demonstrated that honey inhibits apoptosis in rat testicular germ cells [23]. Other studies have demonstrated the antioxidant effect of honey on pyramidal cell proliferation at CA1, CA2, CA3, and dentate gyrus areas of the hippocampus, thereby improving short-term and long-term memory [47]. Other studies have illustrated the beneficial effect of honey in improving memory and cognitive functions of the central nervous system [48]. In another study by Chepulis et al., it has been shown that honey may ease anxiety and improve Spatial Memory in adults [49]. Honey, as an antioxidant, has been traditionally used as a medicine by physicians and Ibn Sina in his book “Ghanoon” has recommended honey in the treatment of various diseases, and to improve the overall health conditions in general because of its antioxidant and antimicrobial properties [50]. Honey contains both enzymatic and nonenzymatic antioxidants such as catalase, ascorbic acid, flavonoids, and alkaloids [51–53]. The combined effect of these antioxidants prevents the oxidation of polyunsaturated fatty acids [51] and lipoproteins [54]. As discussed earlier, hyperglycemia and increasing oxidative stress have been directly linked to neurological complications associated with diabetes [55]. Studies have also shown that type 1 diabetes increases the oxidative stress in the hippocampus of rats [15, 56] leading to the generation of ROS, which attacks and damages the nerve cell membrane and alters mitochondrial membrane permeability, resulting in neuronal loss. Honey can combat damage to the cell membranes by neutralizing the free radicals. In general, it can be concluded from this study that following hyperglycemia due to type 1 diabetes induced by STZ in rats, neuronal damages occur in the hippocampus of rats, and the treatment with insulin, honey, alone, and the combination of honey and insulin can inhibit the progress of neuronal damages in the hippocampus of diabetic rats.

## Figures and Tables

**Table 1:** Characteristic parameters of the control and STZ-diabetic rats.

Parameters	Groups				
	C Mean ± SD	D Mean ± SD	I Mean ± SD	H Mean ± SD	I + H Mean ± SD
Initial body weight (g)	271 ± 52	257 ± 35	235 ± 22	215 ± 17	205 ± 19
Final body weight (g)	315 ± 32	205 ± 12*	259 ± 15.2**	225 ± 11.2	251 ± 17**
Initial blood glucose (mg/dL)	105 ± 7	349.5 ± 54	512 ± 35	574 ± 17	498 ± 25
Final blood glucose (mg/dL)	109 ± 5	512 ± 32*	110 ± 12**	413 ± 29	116 ± 9.5**

C: the control group; D: the untreated diabetic group; I: the diabetic rats treated with insulin; H: rats treated with honey; I + H: diabetic rats treated with insulin and honey simultaneously. *P < 0.01 versus control group (C); **P < 0.01 versus diabetic group (D).

**Table 2:** The effects of insulin and honey on hippocampal neuronal cell viability (trypan blue assay).

Groups	C	D	I	H	I + H
Cellular viability	13506667 ± 5294792^$^	3081667 ± 83321.21*	8369167 ± 1018764^#$^	7776083 ± 1125909^#^	9216917 ± 588563^#$^

C: the control group; D: the untreated diabetic group; I: the diabetic rats treated with insulin; H: rats treated with honey; I + H: diabetic rats treated with insulin and honey simultaneously. *P < 0.01 versus control group (C); ^#^ *P* < 0.001 versus diabetic group (D). ^$^Trypan blue assay.
